# Effect of Biochar Dosage and Fineness on the Mechanical Properties and Durability of Concrete

**DOI:** 10.3390/ma16072809

**Published:** 2023-03-31

**Authors:** Yifu Ling, Xionghua Wu, Kanghao Tan, Zhenjie Zou

**Affiliations:** 1 Key Laboratory of Disaster Prevention and Engineering Safety of Guangxi, School of Civil Engineering and Architecture, Guangxi University, Nanning 530004, China; 2 Guangxi Highway Detection Co., Ltd., Nanning 530004, China

**Keywords:** biochar, cement-based materials, fineness, anti-carbonation, chloride ion resistance, mechanical strength

## Abstract

Biochar (BC), a byproduct of agricultural waste pyrolysis, shows potential as a sustainable substitute material for ordinary silicate cement (OPC) in concrete production, providing opportunities for environmental sustainability and resource conservation in the construction industry. However, the optimal biochar dosage and fineness for enhancing concrete performance are still unclear. This study investigated the impact of these two factors on the mechanical and durability properties of biochar concrete. Compressive and flexural strength, carbonation resistance, and chloride ion penetration resistance were evaluated by varying biochar dosages (0%, 1%, 3%, 5%, 10%) and fineness dimensions (44.70, 73.28, 750, 1020 μm), with the 0% dosage serving as the control group (CK). The results showed that the addition of 1–3 wt% of biochar could effectively reduce the rapid carbonation depth and chloride diffusion coefficient of concrete. The compressive and flexural strength of BC concrete initially increased and then decreased with the increase in biocarbon content, BC with a fineness of 73.28 μm having the most significant effect on the mechanical strength of concrete. At the dosage of 3 wt%, BC was found to promote the hydration degree of cement, improving the formation of cement hydration products. These findings provide valuable insights for the development of sustainable and high-performance cement-based materials with the appropriate use of biochar as an additive.

## 1. Introduction

Cement is the most widely used engineered building material in modern economic construction, and it plays a central role in this industry [1]. However, the use of cementitious materials in concrete comes with major issues of energy consumption and environmental pollution [2,3]. Research has shown that the production of one ton of cement emits about 0.88 tons of CO_2_, contributing to 7.5% of global CO_2_ emissions and causing a significant impact on global warming [4]. Hence, it is crucial to develop a cementitious composite material that exhibits excellent mechanical properties, environmental sustainability, and economic feasibility.

Biochar is a kind of biomass material produced by pyrolysis of waste biomass at a high temperature under anoxic conditions [5]. Incorporating pulverized biochar particles into cement-based materials can improve their mechanical and durability properties through densification and pore-blocking mechanisms, resulting in both environmental and economic benefits [6,7,8]. Although research on the application of biochar in cementitious materials dates back to the 1980s, significant progress has only been made in the past decade. For example, Qin et al. [9] found that the incorporation of less than 6.5% biochar into pervious concrete can enhance its compressive and splitting strength by gradually releasing water stored in the biochar pores, promoting cement hydration and internal solidification. Gupta et al. [10] found that adding 1–2 wt% biochar can adjust the free water-cement ratio in the cement base, thereby enhancing the mechanical properties, ductility, and durability of cement mortar and concrete by 20% and 50%, respectively, compared to the control group. Gupta et al. [11,12] also found that adding 1% BC to cement mortar resulted in 6.95% porosity, which was 11% lower than the control (7.82%). When incorporating more than 5%, the porosity of cement specimens of the same age (7.99%) was only 2% higher than the control. However, the excessive amounts of BC content caused agglomeration, which resulted in the formation of more pores and cracks in the mortar [13]. Other studies have also reported that incorporating biochar can significantly improve the flexural and compressive strength of ordinary concrete and recycled aggregate concrete [14,15]. Additionally, the unique porous nature of biochar contributes to its large surface area, which regulates the effective water content and provides a site for internal solidification and precipitation of hydration products in cement slurry, providing additional advantages over conventional additives [16,17].

Although using biochar in cement-based composites offers many advantages, the impact of biochar fineness on concrete strength development and durability remains inadequately studied. In the existing body of literature, there is a prevailing consensus that the incorporation of fine BC into cement production results in improved hydration reactions and mechanical strength compared to the use of coarse BC [18,19]. Such a viewpoint is corroborated by the findings of Dixit et al. [20], which posited that finely grained fillers yield more substantial benefits with respect to cement hydration and filling than their coarse counterparts [21]. However, it is essential to note that the benefits of reducing the carbon footprint due to the use of biochar may be negated by the energy-intensive process required to grind the particles into finer sizes. Therefore, investigating whether fine biomass coke particles significantly contribute to the enhancement of concrete properties compared to relatively coarse particles is crucial. This comparison will enable a determination of the necessity for additional grinding.

In this study, waste wood was used as the raw material to prepare biochar (BC). Four different fineness levels of BC were produced by grinding with a grinder for 20 s, 30 s, 1 min, and 2 min, respectively, and were used to prepare biochar concrete with varying BC content. The effects of BC on the anti-carbonation and anti-chlorine ion permeability, as well as the compressive and flexural properties of concrete, were investigated. The mechanism underlying the performance change of biochar concrete was analyzed, and the relationship between fineness and BC content is discussed. The findings of this research are expected to contribute to further studies of the properties of cement composites containing BC and the wider use of environmentally friendly BC as a supplementary cementitious material (SCM) to reduce agricultural waste.

## 2. Materials and Methods

### 2.1. Materials

The cement used in this experiment was P.O.42.5 grade ordinary silicate cement from the Conch brand, and its physical properties are shown in Table 1. The fine aggregate employed in the tests was medium sand with a fineness modulus of 2.9 and a maximum particle size of 5 mm. The coarse aggregate utilized was basalt gravel with fineness ranging from 5 to 20 mm and an apparent density of 2750 kg/m^3^. All the materials were procured from the Nanning Conch cement sales store.

### 2.2. Preparation of Biochar

**Figure 1:**
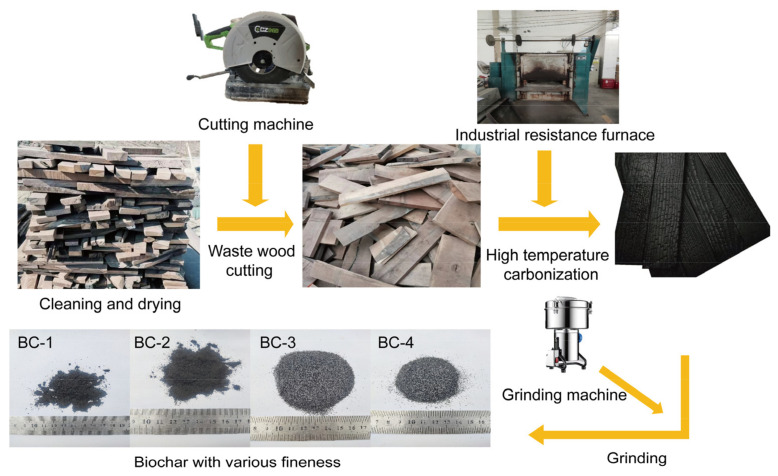
Flow chart of biochar production.

In this study, we chose waste wood as a raw material for the preparation of BC. First, the collected four kinds of raw materials were dried and put into a sealed container and then put it into a muffle furnace, heated to 500 °C for cracking at a heating rate of 20 °C/min under anoxic conditions, and kept at a constant temperature for 2 h to completely carbonize them. The BC obtained by pyrolysis at high temperatures was naturally cooled to room temperature. Then, it was removed from the muffle furnace and crushed with a pulverizer for 20 s, 30 s, 1 min, and 2 min, respectively, to obtain four kinds of BC with different fineness levels, which were placed in sealed bags for later use. The specific production process is shown in Figure 1.

**Figure 2:**
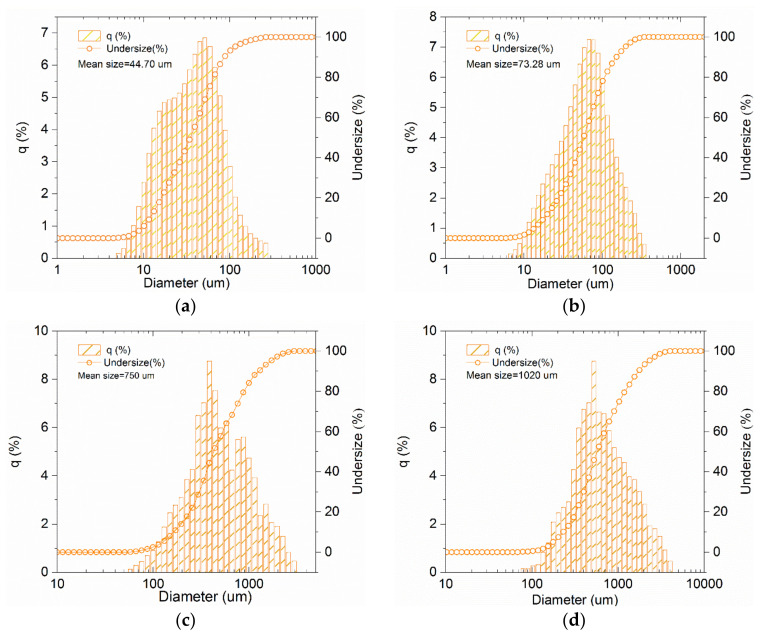
The fineness distribution of BC; (**a**) BC-1, (**b**) BC-2, (**c**) BC-3, and (**d**) BC-4.

The fineness distribution of BC was measured using the LA-960 A laser fineness analyzer from HORIBA, Ltd. in Kyoto, Japan, which is capable of measuring fineness ranging from 10 nm to 5000 μm. Figure 2 shows the fineness distribution of the four biochar samples, labeled as BC-1, BC-2, BC-3, and BC-4, with average fineness levels of 44.70, 73.28, 750, and 1020 μm, respectively.

### 2.3. Design and Preparation of Specimens

BC was used to replace cement in equal amounts at biochar dosages of 0%, 1%, 3%, 5%, and 10% by weight and a water-to-binder (w/b) ratio of 0.45, as presented in Table 2. Concrete test blocks were produced using a combination of manual mixing, mechanical mixing, and plate vibration. The cement and biochar were first pre-mixed manually using a gray shovel until a uniform color was achieved, and then they were added to the JS2000C concrete mixer for mixing. The mortar mix was then poured into appropriate molds in three layers and vibrated using a flat vibrator to ensure even filling and sufficient compaction. After pouring, the concrete was placed in a curing room and maintained for 24 ± 0.5 h before being transferred to a standard curing room (temperature: 20 ± 2 °C; humidity: 95 ± 1.5%) for 6 and 27 days.

### 2.4. Test Methods

Table 3 shows the performed tests, as well as the relevant standards. All the tests used at least three samples.

**Figure 3:**
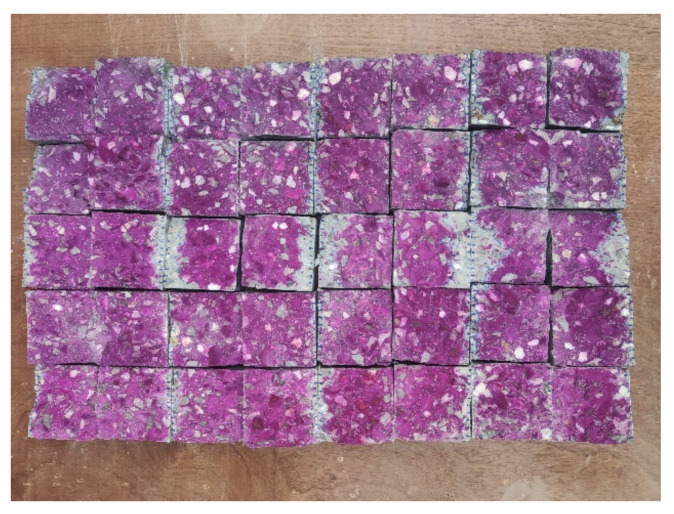
Specimens for carbonation depth testing.

According to GB/t 50082-2009 [22], the carbonation depth of concrete specimens was tested. Concrete specimens were subjected to standard curing for 28 days and then placed in a carbonation box, with minimum spacing of 50 mm between each specimen. The carbonation box environment was maintained per the following conditions: 20 ± 3% carbon dioxide concentration, 20 ± 5 °C temperature, and 70 ± 5% humidity. After seven and 28 days of carbonation curing, the specimens were removed and split from the middle of the specimen, and phenolphthalein test solution was applied to the section of the specimen to determine the carbonation depth, as shown in Figure 3.

To perform the chloride ion rapid penetration test of concrete specimens, the anode and cathode were filled with 0.3 mol/L NaOH solution and 3% NaCl solution, respectively, and the direct current power supply voltage was set at 25 V. The newly cracked section was then sprayed with silver nitrate solution, and the test duration was set to 48 h. The chloride ions infiltrated into the sample reacted with the silver nitrate, resulting in the formation of white precipitate, from which the penetration depth and chloride ion diffusion coefficient Dnssm (10–12 m^2^/s) could be determined. The chloride ion diffusion coefficient is calculated by Equation (1):

(1) $$D_{nssm}=\frac{0.0239(273+T)L}{(U-2)t}(x_{d}-0.0238\sqrt{\frac{(273+T)Lx_{d}}{U-2}})$$

where: *D_nssm_* is the non-stationary migration diffusion coefficient (10^−12^ m^2^/s); *T* is the average value of the initial and final temperatures in the anode electrolyte solution (°C); *U* is the absolute value of the applied voltage (V); *L* is the thickness of the sample (mm); *t* is the test duration (h); and *x_d_* is the average penetration depth (mm) at the seven locations measured.

The XRD experiments were performed using a Rigaku D/MAX 2500V X-ray diffractometer from Rigaku Corporation in Kyoto, Japan. Concrete samples were ground into a uniform fine powder using a mortar and pestle, and an appropriate amount of sample powder was placed on the carrier plate before being fed into the scanning bin. The instrument parameters were set as follows: the scan step was 0.02°, the scan range was 10°–80°, and the tube voltage and tube current were 40 kV and 150 mA, respectively. Additionally, the microscopic morphology of the biochar concrete with different incorporation levels was characterized using the fourth-generation Phenom Pro benchtop scanning electron microscope from Nanoscience Instruments in Eindhoven, Netherlands.

## 3. Results and Discussion

### 3.1. Effect on the Carbonation Depth of Concrete

**Figure 4:**
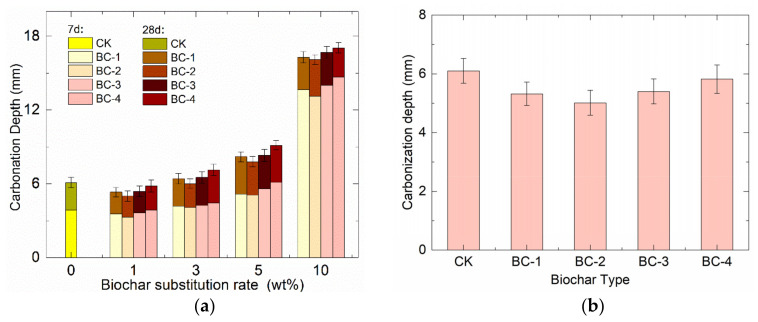
Relationship between: (**a**) biochar dosage and carbonization depth; and (**b**) different types of biochar and chloride diffusion coefficients at 1% dosage.

Figure 4 shows the different trends in the rapid carbonation depth of biochar concrete with biochar dosage and fineness. At low biochar dosages (<3 wt%), biochar had an inhibitory effect on the rapid carbonation reaction of concrete. The most significant effect was observed at the 1% biochar dosage, with which the 28-day carbonation depth of biochar concrete with four different fineness levels (BC-1, BC-2, BC-3, and BC-4, ranging from fine to coarse) was reduced by 12.8%, 17.9%, 11.5%, and 4.6%, respectively, compared to CK. This outcome occurred because the pores of biochar can store water, which is released to reduce the water loss of the specimen during curing, thus maintaining the internal moisture of the concrete and promoting its full hydration and structural densification. Additionally, biochar can adsorb CO_2_ from the air, reducing the CO_2_ concentration on the concrete surface and delaying the carbonation reaction [24,25].

However, when the biochar dosage exceeded 5%, the biochar had a catalytic effect on the rapid carbonation reaction of the concrete because the low activity of the biochar itself and its loose texture could reduce the binding of the cement matrix and overall compactness of the concrete. This outcome, in turn, increased the porosity and permeability of the concrete, making it easier for CO_2_ to penetrate and accelerate the carbonation process [26,27]. It should be noted that, at the same dosage, the different fineness levels of biochar exhibited varying effects on the carbonation reaction. Finer particles tended to retain more water, resulting in more capillaries in the concrete when released later, explaining the different impacts on the carbonation reaction.

### 3.2. Effect on the Chloride Diffusion Coefficient of Concrete

**Figure 5:**
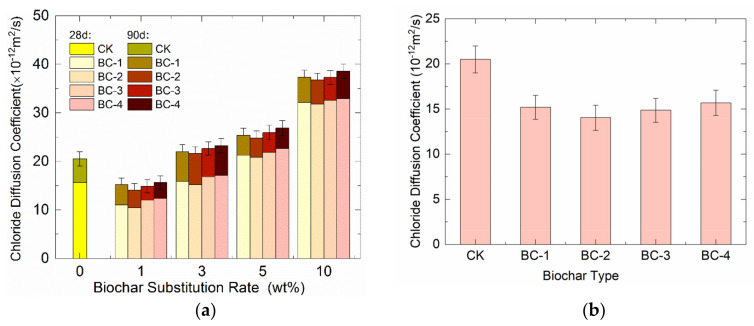
Relationship between (**a**) biochar dosage and chloride diffusion coefficient; and (**b**) the chloride ion diffusion coefficient of biochar concrete and different fineness at 1% dosage.

Figure 5 shows the impact of biochar dosage and fineness on the chloride diffusion coefficient of biochar concrete. As shown in Figure 5a, the chloride diffusion coefficient of concrete gradually increased with the increase in biochar dosage. This outcome can be attributed to the carbonation of biochar mixed mortar producing stable carbonation products, leading to the formation of a dense structure that prevents the diffusion of chloride ions. This outcome, in turn, enhances the resistance to chloride ion attack [28]. Additionally, a small amount of biochar can physically adsorb chloride ions [29]. However, when the biochar content is too high, the content of the cement hydration product Ca(OH)_2_, due to its microfiller effect and limited volcanic ash reaction, is insufficient, resulting in an underdeveloped volcanic ash reaction. As the dosage of biochar increases and the amount of cement decreases, the total amount of cement hydration products decreases, leading to insufficient adhesion. Furthermore, as biochar is a high porosity material, increasing its admixture will increase the internal porosity of concrete [26]. Thus, with an increase in biochar admixture, there is a phenomenon in which the chloride ion penetration resistance of concrete decreases instead of increasing.

Figure 5b shows that the chloride diffusion coefficient of concrete first decreases and then increases with the increase in biochar fineness at the same dosage. When the biochar dosage was 1 wt%, the 90-day chloride diffusion coefficients of concrete with four different biochar fineness levels (BC-1, BC-2, BC-3 and BC-4) were 15.2, 14.04, 14.86 and 15.68 × 10^−12^ m^2^/s, respectively. This outcome indicates that BC-2 had a more significant effect on reducing the chloride diffusion coefficient of concrete than other fineness levels at dosages less than 1 wt% because coarse biochar particles absorb more water than fine biochar particles at the same water-cement ratio, resulting in a lower local water-cement ratio of concrete containing coarse biochar particles. Furthermore, coarse biochar particles release water at a later stage and create more capillary pores inside the concrete, increasing the permeability of the concrete and enhancing the chloride diffusion.

**Figure 6:**
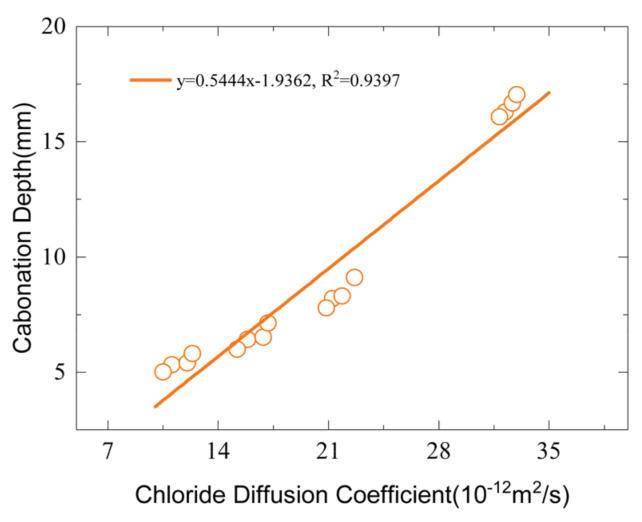
Relationship between carbonization depth and chloride diffusion coefficient.

Figure 6 presents the relationship between carbonation depth and chloride diffusion coefficient for this study. It can be seen that there is a good relationship between different transport mechanisms, such as gas diffusion and ion diffusion. This relationship implies that carbonation and chloride ingress are coupled processes that affect each other’s rate and extent. Carbonation reduces the pH of concrete and decreases its chloride binding capacity, increasing the free chloride concentration and enhancing the chloride diffusion [30]. On the other hand, chloride ions can lower the carbonation depth by increasing the alkalinity of pore solution and inhibiting the dissolution of calcium hydroxide [30,31].

### 3.3. Effect on Compressive and Flexural Strength of Concrete

**Figure 7:**
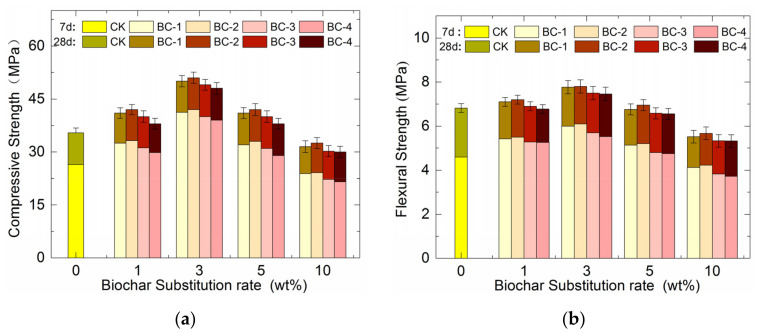
Mechanical strength of concrete with different fineness levels and biochar dosages: (**a**) compressive strength; (**b**) flexural strength.

Figure 7a shows the effects of biochar fineness and dosage on the compressive strength of concrete. The compressive strength of concrete increased and then decreased with increasing biochar fineness levels at the same dosage, but this change is not significant. However, as the biochar dosage increased, the compressive strength also tended to increase and then decrease. Specifically, when the biochar dosage was 3 wt%, the compressive strength of the concrete reached a maximum value, increasing by 15.66%, 18.48%, 12.83%, and 7.19% for BC-1, BC-2, BC-3, and BC-4, respectively. The most significant increases were observed in BC-1 and BC-2, and the effect was found to be more pronounced with the extension of curing time. This outcome was attributed to the volcanic ash activity and filler effect of the fine biochar, which improved the pore network of the concrete, providing good internal curing conditions for cement hydration reactions during curing, ultimately increasing the hydration and strength of the concrete. This finding was consistent with previous research by Wang [32]. When the dosage of biochar exceeded 5 wt%, the compressive strength of biochar concrete began to show a decreasing trend but remained higher than CK. However, when the dosage of biochar exceeded 10 wt%, the compressive strength of biochar concrete was lower than CK. For instance, the 28-day strength of BC-1, BC-2, BC-3, and BC-4 concrete decreased by 11.14%, 8.32%, 14.7%, and 15.33%, respectively, compared to CK. Therefore, the negative effects of biochar on concrete at high admixture levels outweighed the positive effects.

The impact of biochar on the flexural strength of concrete exhibited a similar pattern to its effect on compressive strength, as illustrated in Figure 7b. However, the key difference was that, while biochar does have a limited capacity to increase the flexural strength of concrete, it also leads to a significant reduction in this property. Specifically, at lower admixture levels (1 wt% and 3 wt%), the flexural strength of concrete showed a slight increase, whereas at higher admixture levels (5 wt% and above), it was lower than that of the control group and decreased with increasing admixture and fineness. For instance, in the 28-day flexural strength test, the strength of BC-4 concrete (10 wt%, maximum fineness) was only 77.97% that of the unadulterated biochar concrete, whereas the strength of BC-1 concrete (10 wt%, minimum fineness) was 80.9%. The most significant reduction, amounting to 22.03%, was observed in BC-4 concrete. This phenomenon can be attributed to the physical properties of biochar. On the one hand, BC’s high porosity and low Young’s modulus result in a reduction in the Young’s modulus of the concrete [33]. On the other hand, the contribution of biochar to the strength of concrete specimens is small or negligible compared to that of other materials, such as gravel and sand, due to its low hardness [34]. When subjected to forces, the larger fineness level of biochar creates greater voids in the concrete, leading to more significant stress concentrations and reduced effective bearing area.

### 3.4. SEM Analysis

**Figure 8:**
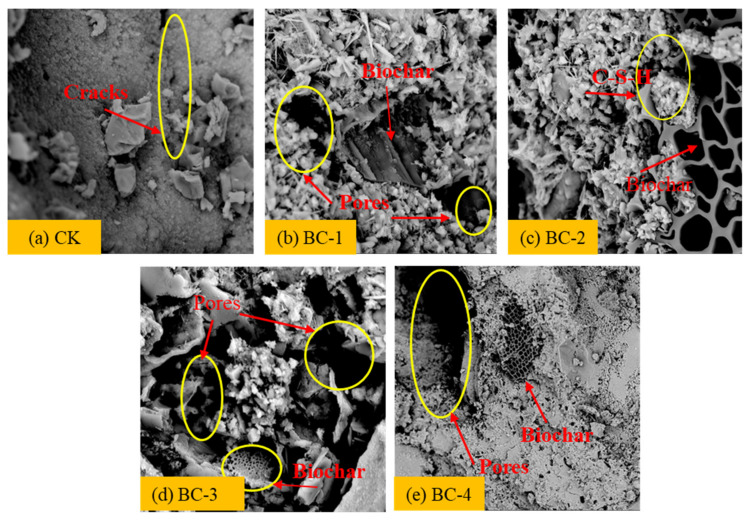
SEM images of concrete with different fineness levels of biochar with 3% content.

According to the above experimental results, the mechanical and durability properties of concrete were improved when the biochar content was 3 wt%. To further analyze the microstructural changes, SEM images of biochar concrete at 3 wt% dosage for 28 days were obtained, as shown in Figure 8. It can be seen that, without the addition of biochar, the hydration products in the concrete were mainly granular C-S-H crystals with many micro-pores between them and a loose structure. Cracks were easily formed at the interfacial transition zone (ITZ) and failed to effectively connect and fill (Figure 8a). The addition of biochar caused a micro-filler effect and provided more nucleation sites for cement hydration.

When BC-1 was added, although various hydration products interwoven into a dense and uniform structure were locally visible at the interface, biochar aggregated together due to van der Waals forces and could not play a cementing role to produce C-S-H crystals and other hydration products, forming larger pores and cracks around it. A small amount of flocculent C-S-H crystals were also scattered on the surface. These pores and cracks were the main reasons for the decrease in macroscopic mechanical properties of concrete specimens when excessive biochar was added (Figure 8b). When BC-2 was added, a large amount of fibrous AFt was visible on the entire surface of the cement paste interface and was evenly distributed with biochar. The added biochar was wrapped by hydration products and filled in the internal pores of the concrete, forming a continuous overall structure without larger pores or larger hydrated crystals (Figure 8c). When BC-3 and BC-4 were added, due to their larger fineness levels, they increased the number of local pores and cracks around ITZs and also caused more pores and cracks inside cement paste bodies, resulting in adverse effects, such as local stress concentrations, reduced effective bearing area, etc., leading to lower macroscopic mechanical properties of concrete (Figure 8d,e).

### 3.5. XRD Analysis

**Figure 9:**
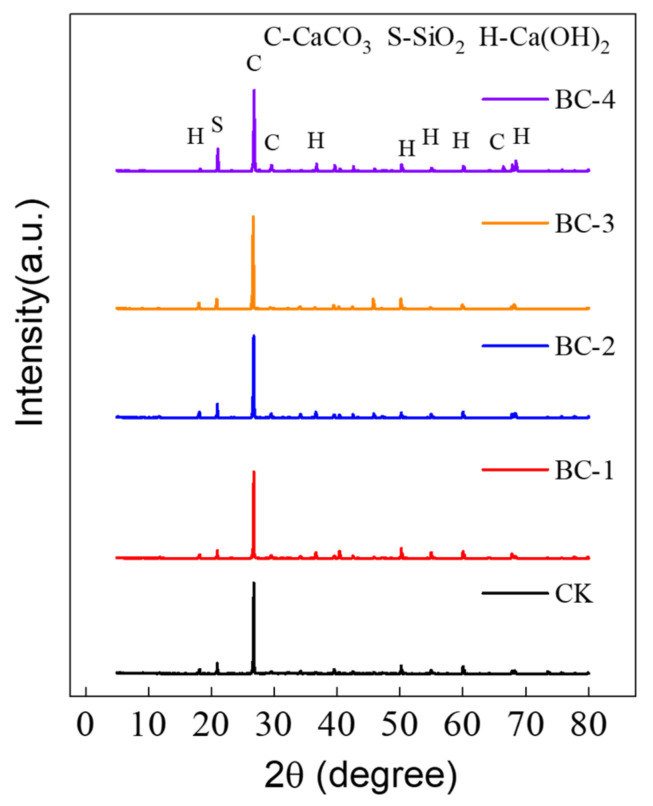
XRD image of biochar concrete with different fineness levels at 3% dosage.

To further investigate the impact of biochar fineness on cement hydration, X-ray analysis was conducted on 28-day concrete specimens with a 3% biochar dosage. The main mineral composition of each group of specimens is presented in Figure 9. The concrete hydration products’ crystals with the most prominent diffraction peak intensities were CaCO_3_, SiO_2_, and Ca(OH)_2_. Their characteristic peaks changed with variations in biochar fineness. As the biochar fineness decreased, the intensity of CaCO_3_ diffraction gradually decreased, while the intensity of SiO_2_ and Ca(OH)_2_ diffraction initially decreased and then increased.

These findings suggest that biochar modifies the cement hydration process by influencing the formation and transformation of hydration products. When added in appropriate quantities, Ca(OH)_2_ reacts with SiO_2_ based on biochar as a carrier [35]. This process consumes a considerable amount of the lower-strength Ca(OH)_2_ and transforms it into a higher-strength C-S-H cementitious gel, which fills the concrete pores and increases its strength, better explaining the concrete’s compressive strength peak. However, when BC-3 and BC-4 were added, the secondary reaction of the hydration product Ca(OH)_2_ was weakened or even prevented by the adsorption of some of the free water onto the biochar. Consequently, there were increases in Ca(OH)_2_ and SiO_2_ content and a reduction in the C-S-H gel produced, leading to a reduction in the biochar concrete’s strength development.

## 4. Conclusions

This paper investigated the four different fineness levels of biochar made from waste wood as a raw material and their effects on the anti-carbonation properties, chloride ion resistance, and compressive and flexural strength of cement materials after mixing with biochar. Based on the experimental results, the following conclusions were drawn:

1. Increasing biochar fineness initially led to a decrease in rapid carbonation depth and chloride diffusion coefficient, followed by an increase. Adding 1–3% biochar resulted in the rapid carbonation depth and chloride diffusion coefficient of concrete decreasing by a maximum of 17.9% and 32%, respectively.
2. The compressive and flexural strength of concrete increased and then decreased as the replacement rate of BC increases. When the content was less than 5%, the strength of biochar concrete was not lower than that of the control group. Of all the biochar fineness levels tested, BC-2 exhibited the most significant improvement in the strength of concrete.
3. SEM and XRD analyses demonstrated that the addition of biochar enhanced the microstructure of concrete by facilitating additional nucleation sites for cement hydration and filling internal pores, ultimately leading to a more uniform and dense composition.

In conclusion, the addition of biochar made from waste wood could effectively improve the anti-carbonation properties, chloride ion resistance, and mechanical strength of cement-based materials. The results of this study provide valuable insights for the development of sustainable construction materials with improved performance. 

## Figures and Tables

**Table 1:** Chemical composition and physical properties of cement.

Chemical Composition (%)		Physical Properties	
CaO	63.57	Specify Gravity (g/cm^3^)	3.16
SiO_2_	20.03	Blaine Specific Surface (cm^2^/g)	4650
Al_2_O_3_	6.26	Initial Setting (min)	165
Fe_2_O_3_	2.59	Final Setting (min)	254
MgO	2.68	Compressive Strength (MPa)	-
Na_2_O and K_2_O	0.95	3 d	20.8
MnO	0.33	28 d	47.6
SO_3_	2.01	Flexural Strength (MPa)	-
P_2_O_5_	-	3 d	4.4
LOI	1.80	28 d	8.6

**Table 2:** The material mix ratios of the mortar specimens made.

Sample	Cement (kg/m^3^)	Sand (kg/m^3^)	Coarse Aggregate (kg/m^3^)	Water (kg/m^3^)	Biochar (kg/m^3^)	Biochar Alternative Cement Ratio (wt%)
CK	433	611.05	1134.82	195	0	0
BC-1/2/3/4	428.67	611.05	1134.82	193.05	4.33	1
	420.01	611.05	1134.82	189.15	12.99	3
	411.35	611.05	1134.82	185.25	21.65	5
	398.36	611.05	1134.82	179.4	34.64	8

**Table 3:** Test program and relevant standards.

Property	Standard Followed	Remarks
Concrete carbonation depth	GB/t 50082-2009 [22]	The sample size was 150 × 150 × 150 mm^3^
Concrete chloride diffusion coefficient		
Compressive strength	GB/T 50081-2016 [23]	
Flexural strength		The flexural strength was tested using a four-point loading method on beam specimens with dimensions of 100 × 100 × 400 mm^3^

## Data Availability

Some or all data that support the findings of this study are available from the corresponding author upon reasonable request.
